# Variations of high frequency parameter of heart rate variability following osteopathic manipulative treatment in healthy subjects compared to control group and sham therapy: randomized controlled trial

**DOI:** 10.3389/fnins.2015.00272

**Published:** 2015-08-04

**Authors:** Nuria Ruffini, Giandomenico D'Alessandro, Nicolò Mariani, Alberto Pollastrelli, Lucia Cardinali, Francesco Cerritelli

**Affiliations:** ^1^Research Department, Accademia Italiana Osteopatia TradizionalePescara, Italy; ^2^Clinical-based Human Research Department, Centre for Osteopathic Medicine CollaborationPescara, Italy

**Keywords:** osteopathic medicine, heart rate variability, high frequency, healthy subjects, smoking, autonomic nervous system, parasympathetic effect

## Abstract

**Context:** Heart Rate Variability (HRV) indicates how heart rate changes in response to inner and external stimuli. HRV is linked to health status and it is an indirect marker of the autonomic nervous system (ANS) function.

**Objective:** To investigate the influence of osteopathic manipulative treatment (OMT) on cardiac autonomic modulation in healthy subjects, compared with sham therapy and control group.

**Methods:** Sixty-six healthy subjects, both male and female, were included in the present 3-armed randomized placebo controlled within subject cross-over single blinded study. Participants were asymptomatic adults (26.7 ± 8.4 y, 51% male, BMI 18.5 ± 4.8), both smokers and non-smokers and not on medications. At enrollment subjects were randomized in three groups: A, B, C. Standardized structural evaluation followed by a patient need-based osteopathic treatment was performed in the first session of group A and in the second session of group B. Standardized evaluation followed by a protocoled sham treatment was provided in the second session of group A and in the first session of group B. No intervention was performed in the two sessions of group C, acting as a time-control. The trial was registered on clinicaltrials.gov identifier: NCT01908920.

**Main Outcomes Measures:** HRV was calculated from electrocardiography before, during and after the intervention, for a total amount time of 25 min and considering frequency domain as well as linear and non-linear methods as outcome measures.

**Results:** OMT engendered a statistically significant increase of parasympathetic activity, as shown by High Frequency power (*p* < 0.001), expressed in normalized and absolute unit, and possibly decrease of sympathetic activity, as revealed by Low Frequency power (*p* < 0.01); results also showed a reduction of Low Frequency/High Frequency ratio (*p* < 0.001) and Detrended fluctuation scaling exponent (*p* < 0.05).

**Conclusions:** Findings suggested that OMT can influence ANS activity increasing parasympathetic function and decreasing sympathetic activity, compared to sham therapy and control group.

## Introduction

Heart rate, one of the most variable organism's phenomena, corresponds to a number of heart beats per minute. Heart rate variability (HRV) is the variation of the consecutive RR interval period (Aa.Vv., 1996; Kara et al., 2003; Freeman et al., 2006), where *R* is a point corresponding to the QRS complex peak of the electrocardiogram wave, related to left ventricle depolarization. Its variability, depending on metabolic (inner) and environmental (external) stimuli (Caruana-Montaldo et al., 2000; Henley et al., 2008; Routledge et al., 2010), is responsible of maintaining homeostasis. HRV can be influenced by several factors, such as intrinsic variability of sinoatrial node (Ponard et al., 2007), neurohormonal hematic fluctuation (Galetta et al., 2008), circadian rhythm (Huikuri et al., 1990; Bonnemeier et al., 2003; Cavallari et al., 2010), and autonomic nervous system (ANS) activity.

HRV is considered an indirect proxy of ANS function (Aa.Vv., 1996; Porta et al., 2001; Malliani and Montano, 2002; Pumprla et al., 2002; Freeman et al., 2006; Kemp et al., 2012), as parasympathetic and sympathetic activity regulates cardiac rate and inotropism. These branches respectively lead to a reduced or increased efficiency of the cardiovascular pump, modifying, therefore, HRV parameters (Aubert et al., 2003; Guyton and Hall, 2006). To quantify the sympathetic and parasympathetic activities, several HRV indices and methods are used. The most common are the frequency domain indices—low frequency (LF) and high frequency (HF)—and the ratio between LF/HF.

Among the external factors able to influence HRV-values, smoking has been demonstrated to be particularly strong (Minami et al., 1999; Pope et al., 2001). A study conducted in 1999 by Minami et al. investigated the effect of smoking cessation on HRV and other cardiovascular indices (Minami et al., 1999). Results showed that smoking cessation significantly decreased the high frequency (HF) component throughout a 24-h period, indicating that in habitual smokers, parasympathetic nervous system is altered. HRV parameters have been linked, also, to health status (Karemaker and Lie, 2000).

In addition to traditional medicine, also complementary and alternative medicines (CAM) have been starting to use HRV variations to understand how different therapeutic approaches, like homeopathy (Mishra et al., 2011), massage (Lee et al., 2011), acupuncture (Haker et al., 2000; Li et al., 2003; Litscher, 2009, 2010; Arai et al., 2011; Anderson et al., 2012; Huang et al., 2012; Litscher et al., 2012), auricular acupuncture (Gao et al., 2012) can influence health status.

Among CAM, osteopathic medicine is a form of drug-free non-invasive manual medicine. It relies on manual contact for diagnosis and treatment. Osteopaths use a wide variety of tests to locate the somatic dysfunction (SD) (ICD-101CM Diagnosis Code M99.09-09). Diagnostic criteria are focused on tissue abnormality and tone. Areas of asymmetry, misalignment of bony landmarks and the quality of motion are evaluated. Osteopathic care is based on two phases: structural evaluation and treatment. The structural evaluation aims to locate SDs using a well-established sequence of structural tests. The treatment currently encompasses more than 20 types of manual techniques and has the scope to treat SDs.

In osteopathy, Henley's work (Henley et al., 2008) examined HRV changes in healthy subjects receiving osteopathic manipulative treatment (OMT) compared to control and sham therapy groups after tilt test. Low frequency expressed in normalized unit (_nu_LF) significantly increased in the control and sham groups compared to OMT group when subjects move from horizontal to head-up tilt. After the application of OMT, _nu_LF increased significantly less in the head-up position than in control and sham groups. Contextually high frequency, measured in normalized units, (_nu_HF) was significantly lower in OMT group compared to sham and control. Moreover, OMT group occurred to have a lower LF/HF ratio in the head-up tilt phase after intervention compared to sham and control groups.

In 2013, another osteopathic study (Giles et al., 2013) investigated the effect of sub-occipital decompression (a specific cranium-sacral technique) on HRV indices in healthy subjects, showing an increase of absolute HF and a decrease of LF/HF ratio, compared to sham therapy and control groups. However, several limitations could be pointed out leading to lack of clinical generalizability of findings. Firstly, both research focused on a single session without investigating the long term variations of HRV parameters. Secondly, smokers were not included in Henley's study, while in Giles' one no information was provided about smoking. Finally a single standard, pre-determined technique was administered in both studies not considering usual clinical care procedures. The present trial, therefore, aimed to investigate the effect of OMT on ANS using HRV as a primary outcome measure.

## Materials and methods

Primary outcome of this randomized placebo controlled within subject cross-over single blinded study was to explore the extent to which OMT changed _nu_HF-value, compared to sham therapy and control group. Secondary outcomes were baseline changes in _au_LF, LF/HF ratio and Detrended fluctuation scaling exponent (DFAα1), HF absolute power (_au_HF measured in absolute units; i.e., ms^2^).

### Population

Asymptomatic healthy adults, of either gender, were considered eligible for the study. Inclusion criteria applied were: age between 18 and 45 years; absence of chronic pain or acute symptomatology during the last 72 h before the session; no diagnosed pathology.

Exclusion criteria were: pregnancy; menopause; menstrual flow during the session; alcoholism; alcohol abuse 48 h prior to the session; chronic pain; diagnosis of pathological condition; chronic drug treatment; use of medications and drugs during the last 72 h; use of orthotic devices during the last 3 months; medical history of surgical interventions; last OMT at least 3 months before the session.

Volunteers from different universities were recruited from May 01, 2013 to July 31, 2013 through e-mail, phone, direct contact and leaflet/posters. The latter were posted at Accademia Italiana Osteopatia Tradizionale (AIOT) and at an osteopathic clinical center inviting people to call a phone number managed by the recruitment supervisor.

Informed consent was read and signed by each volunteer at the beginning of the first session. The trial was approved by the institutional review board of AIOT. The trial was registered on clinicaltrials.gov identifier: NCT01908920.

### Experimental protocol

**Figure 1:**
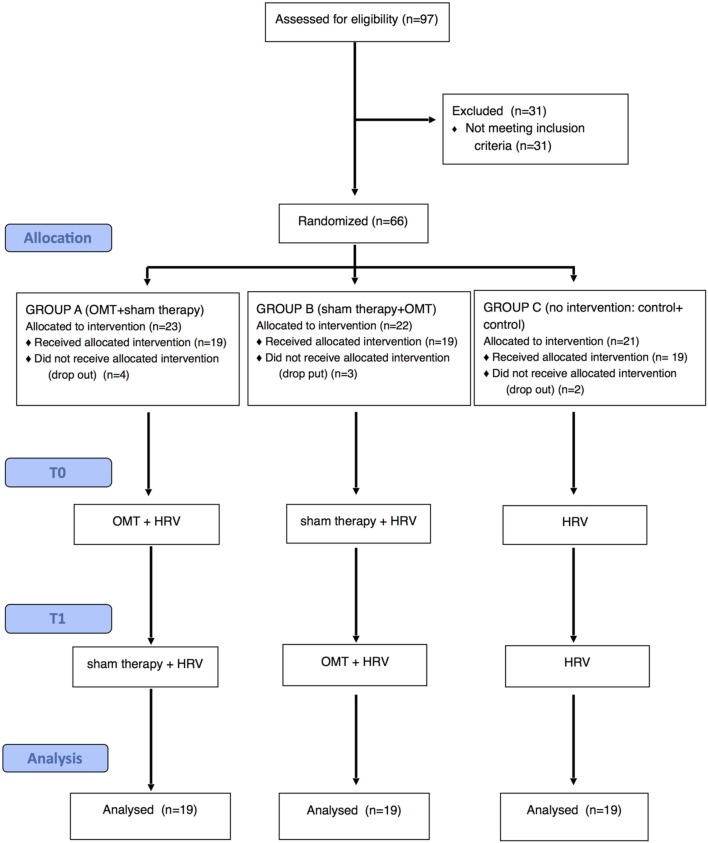
**Flow-chart of the study**.

Subjects were divided and randomized into three groups using “permuted block” randomization method (block size equal to 3), formulated by the statistical software R v.2.15.1 (R Developmental Core Team, 2010) and stratified by smoking. A ternary sequence (0, 1, 2) was used to allocate subjects into groups A–C (Figure 1).

First session could be rescheduled if the subject presented at least one of the following exclusion criteria: acute pain in the last 72 h, assumption of any medicine or drug in the 72 h before the session, menstrual flow during the intervention day, alcohol abuse in the last 48 h. Subjects were considered drop-out in case of nonattendance at the second session.

Subjects allocated to treatment groups A and B received both an OMT and a sham therapy session at different time points on the basis of the cross-over design. At the first session group A received OMT while group B received sham treatment. At the second session group A received sham treatment whilst group B received OMT. Group C was used as time control thus neither treatment nor sham therapy were provided in both two sessions (Figure 1).

Evaluations and treatments were performed in the same room, with stable temperature and humidity, to avoid any influence on ANS activity. Moreover, the 2 weekly sessions were scheduled at the same hour to control for circadian rhythm.

The OMT intervention consisted in a patient's need based treatment, thus no pre-determined protocol was applied. Osteopathic session lasted 25 min, 10 min for evaluation and 15 min for treatment. Techniques used in the present study were left at the discretion of the operator but limited to balance ligamentous techniques, balance membranous techniques and cranio-sacral techniques (Magoun, 1976).

For this trial, sham therapy mimicked the osteopathic care, based, therefore, on structural evaluation and treatment. The sham evaluation overlapped the osteopathic structural evaluation procedure in terms of tests and time. The sham treatment consisted in contacting an established sequence of anatomical areas for 2 min each: right ankle, left knee, right hip, diaphragm, right shoulder, neck, cranium. The operator mentally counted from 120 to 0 for each area to prevent placebo autonomic activation (Meissner, 2011). Sham session lasted 25, 10 min for evaluation and 15 min for treatment.

All interventions were performed by four osteopaths with the same educational curricula assigned randomly to group A or B. Each osteopath was responsible for the same patient from enrollment to the end of the study.

Control group sessions were managed by one operator in charge of data collection, no osteopaths were present.

Osteopaths were blinded to the study design, outcome and HRV data. Two external operators were in charge of randomization and data collection. Both were blinded to the study design and outcome, as well as allocation. All data collected was examined by an external statistician, who was blinded of randomization, allocation and study design. Subjects were blinded to study design, outcome and the type of intervention received.

### Data analyses

Data was collected through a socio-demographic form and a HRV detector system Flexcomp (http://www.righetto.biz/Biofeedback/flexcomp_infiniti.htm). The latter is composed of a plethysmograph put on the third finger of the left hand, two cutaneous conductance detectors on the second and fourth left fingers and two stripes positioned around the thorax and the abdomen to control lung ventilation. R–R intervals were extracted from ECG (256 Hz sample rate) using Physiology Suit software (www.thoughttechnology.com/physsuite) and imported in Kubios software (http://kubios.uef.fi) to calculate all HRV parameters considered in the present trial. RR series of 300 cardiac beats were analyzed. Only one sequence was analyzed in each condition. If more than 300 RR-values were recorded, a sequence of 300 beats was selected randomly inside the period of analysis. If less than 300 RR-values were recorded, all the values were analyzed.

HRV analysis method, based on processing recorded RR intervals, was divided into linear analysis (time and frequency domain) and nonlinear analysis (Aubert et al., 2003). From power spectra (Fast Fourier transformation using Blackman Harris window) of equidistant linear interpolated (4 Hz) tachograms (resampled to 2 Hz) the following frequency domain standard HRV indices were considered for linear analysis: absolute HF and _nu_HF, from 0.15 to 0.4 Hz, is a signal of parasympathetic modulation on heart rate (Aa.Vv., 1996; Berntson et al., 1997; Taylor et al., 1998); absolute LF and _nu_LF component, from 0.04 to 0.15 Hz bandy (Lane et al., 2009). LF/HF ratio is showed to be a reliable marker of ANS balance (Pagani et al., 1986; Malliani et al., 1991). DFAα1 was considered for nonlinear analysis. DFAα1 is a proxy of the parasympathetic function, it is considered more sensitive (Kemp et al., 2010, 2012) and able to catch the long term correlations and complexity in RR interval series (Peng et al., 1993). The DFA method detected long-range correlations between inter-beat intervals separated by several beats by investigating the scaling behavior of the heartbeat fluctuations on different time scales. A detailed description of the DFA algorithm and its underlying theory for the analysis of neuronal oscillations was pertinently presented by Hardstone et al. (2012). A fractal structure of heart rate was quantified by estimating a short-term (a1, short-term fluctuations, obtained from the range 4 ≤ *n* ≤ 16) and a long-term (a2, long-term fluctuations, obtained from the range 16 ≤ *n* ≤ 64) scaling exponent by DFA.

At enrollment, subjects completed the socio-demographic form. For each session, a paradigm was developed consisting of 4 conditions: (1) 5 min no hands-on for baseline assessment; (2) 10 min for either osteopathic or sham evaluation; (3) 15 min for the treatment, hands-on subjects; and (4) 5 final min where no hands contact was provided.

HRV records were performed at (1), (3), and (4).
Subjects were instructed to keep still, eyes closed and in silence during the three recording steps.

### Statistical analysis

Sample size was computed considering a correlation coefficient among repeated measure of 0.15, an effect size of 0.3, a type-one error of 0.05 and a power of 0.80. The final sample *n* = 51 (17 per group) was additionally increased up to 60 to prevent loss of power.

A per protocol analysis was performed in the present trial. Stationary was assessed using the RWS test (Porta et al., 2004), which checks if mean and variance remain constant over *M* patterns. Giving a series of RR, the first step was to use Kolmogorov Smirnov goodness-of-fit test to evaluate the normality of the distribution (*p* < 0.05). If non-normal distribution resulted, a log transformation was applied and normality retested. If data was still non-normally distributed, then it was finally rejected. The next step was to test normality along *M* patterns, which are randomly selected from a set of sequences of length *L* (Porta et al., 2004). To test the stability of the mean an ANOVA was performed, otherwise Kruskal–Wallis test. To test the stability of the variance, Barlett test was used if data was normally distributed otherwise Levene's test using the median was adopted.

A descriptive analysis of the population was performed at the baseline using arithmetic means, medians, standard deviations and standard errors. Univariate statistical tests used chi square test for establishing differences among categorical variables, such as gender, OMT, and smoking. Results were expressed in percentage.

At baseline, One-Way ANOVA was used to analyze continuous variables, such as age, BMI, _nu_HF, _au_HF, _nu_LF, _au_LF, LF/HF ratio, and DFAα1. Tukey *post-hoc* analysis was used to explore any statistical difference resulted from ANOVA.

Mixed effect regression (MER) model considering random effect for osteopath and a fixed effect for period was used to determine dependent variables differences between groups. *Post-hoc* pairwise analysis adjusted by Holm-Bonferroni correction was used to explore any statistical difference resulted from MER. Sensitivity analyses were performed according to smoking and session.

Effect size was computed using Cohen's *d* to show any clinical effect of OMT compared to sham therapy and control group. Statistical significance was based on a probability level at less than 0.05. All analyses were performed using R v 2.15.01.

## Results

Out of 97 volunteers, 66 were included in the study and randomized. Nine subjects dropped out and consequently 57 completed the study (Figure 1). None of the subject enrolled recorded less than 300 cardiac cycles at any study condition.

The RWS revealed a stationarity of mean and variance. At enrollment, One-Way ANOVA did not reveal any statistically significant imbalance among OMT, sham and control, in terms of age, BMI, gender, previous OMT experience, smoking, _nu_HF, _au_HF, _nu_LF, _au_LF, DFAα1, and LF/HF ratio values (Table 1). Among the 38 participants receiving sham and OMT interventions, the percentage of subjects able to correctly guess which treatment they received did not differ (session 1: 16% OMT vs. 21% Sham, *p* = 0.68; session 2: 31% OMT vs. 26% Sham, *p* = 0.72).

**Table 1:** **Baseline values of osteopathic manipulative treatment (OMT), Sham, and Control groups**.

	**OMT**	**Sham**	**Control**	***F***	***p* adj**
Age^*^	26.5 ± 8.8	28 ± 8.1	25.6 ± 8.2	0.46	0.63
BMI^*^	18.9 ± 8.8	18.7 ± 2.4	18.0 ± 3.3	0.46	0.63
Male^$^	10 (53)	14(74)	10 (53)	–	0.31
Previous OMT^$^	5 (26)	8 (42)	8 (42)	–	0.5
Smoking^$^	7 (37)	6 (32)	7 (37)	–	0.93
auHF^*^	1391 ± 1850	1138 ± 650	944 ± 1563	0.92	0.40
nuHF^*^	47.12 ± 26.36	50.85 ± 17.4	46.8 ± 18.5	0.34	0.71
auLF	1138 ± 676	1481 ± 2098	1290 ± 1705	0.89	0.48
nuLF^*^	54.34 ± 26.37	48.99 ± 17.41	53.14 ± 18.52	0.61	0.55
DFAa1^*^	1.05 ± 0.33	0.91 ± 0.19	0.96 ± 0.26	0.55	0.58
LF/HF^*^	2.34 ± 2.6	1.28 ± 1.09	1.87 ± 2.41	0.47	0.63

Numbers in table are:

* mean ± sd

$ n (%). p-values from ^*^ANOVA and $chi square test.

### Primary outcome

**Figure 2:**
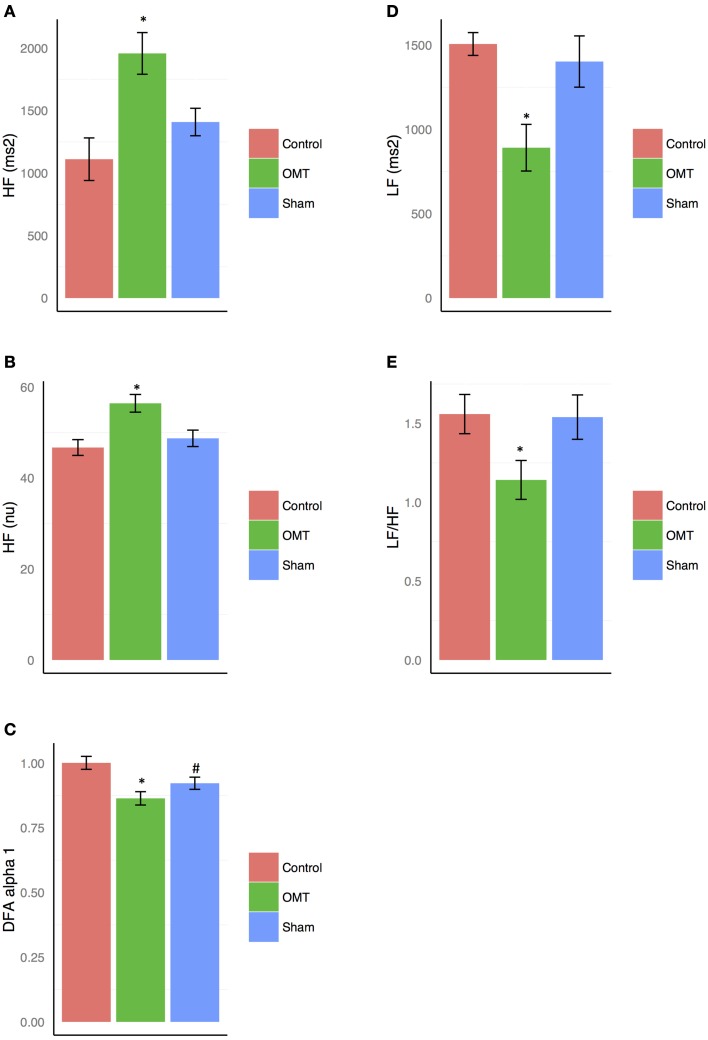
**Heart rate variability (HRV) findings for: high frequency (HF) absolute units (au, A) and normalized units (nu, B); detrended fluctuation scaling exponent (DFAα1, C); low frequency (LF, D); low frequency/high frequency ratio (LF/HF, E)**. Data presented are means ± standard errors of the mean (SEM). ^*^statistically significant differences (*p* < 0.001) in OMT group compared to sham and control groups. # statistically significant decrease of DFAα1 (*p* < 0.05) in sham group compared to control. C, control; O, osteopathic manipulative treatment; S, sham therapy.

MER showed a statistically significant difference between groups on the primary outcome _nu_HF (*p* < 0.001) (Figure 2B). Tukey *post-hoc* analysis revealed that OMT group significantly increased _nu_HF-values compared to sham (*p* < 0.01) and control group (*p* < 0.001). Sham therapy did not show any significant modification of _nu_HF-values compared to control group (*p* = 0.44). OMT had a medium effect size compared to sham therapy (*d* = 0.38) and control group (*d* = 0.49).

### Secondary outcome

#### Low frequency

_au_LF analysis showed a statistically relevant difference between groups (*p* < 0.01) (Figure 2D). Tukey analysis demonstrated that OMT significantly decreased _au_LF-values compared to sham (*p* < 0.05) and control (*p* < 0.001). No similar results were shown for sham and control (*p* = 0.40).

#### Detrended fluctuation scaling exponent (DFAα1)

DFAα1 was statistically different between groups (*p* < 0.05) (Figure 2C). *Post-hoc* analysis showed that OMT significantly decreases DFAα1-values compared to control (*p* < 0.001) but it was almost significant compared to sham therapy (*p* = 0.09). Moreover, sham therapy significantly lowered DFAα1 compared to control group (*p* = 0.05). Effect sizes were small (OMT vs. sham group: *d* = 0.22) and medium (OMT vs. control: *d* = 0.51; sham vs. control: *d* = 0.30).

#### Low frequency/high frequency ratio

Statistically significant decrease of LF/HF ratio was observed between groups (*p* < 0.001) (Figure 2E). Tukey *post-hoc* analysis showed that OMT scored significantly better than sham therapy (*p* < 0.001) and control (*p* < 0.001).

Sham therapy did not show any statistical association compared to control group (*p* = 0.40).

#### High frequency—absolute unit

_au_HF showed statistically significant differences between groups (*p* < 0.01) (Figure 2A). *Post-hoc* analysis demonstrated that in the OMT group a significant increase was revealed compared to sham and control (*p* < 0.001).

### Sensitivity analysis

Sensitivity analyses were performed taking into account smoking and sessions.

**Figure 3:**
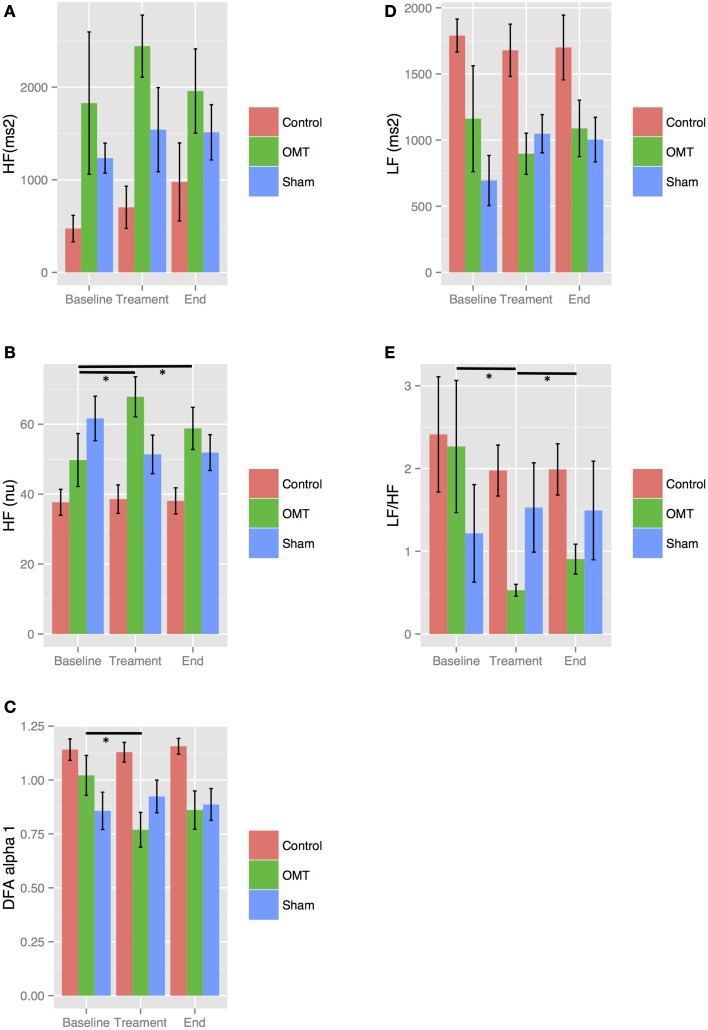
**Sensitivity analysis stratified by smoking**. Numbers are mean ± SEM. HF(au) **(A)**, high frequency absolute units; HF(nu) **(B)**, high frequency normalized unit; DFAα1 **(C)**, detrended fluctuation scaling exponent; LF **(D)**, low frequency; LF/HF **(E)**, low frequency/high frequency ratio. Each figure shows HRV data recorded during the three steps of the each session: baseline (5 min), treatment (15 min), end (5 min). ^*^p < 0.001 from regression mixed effect model. LF and LF/HF ratio showed a relevant increase in OMT group between end and treatment periods.

**Figure 4:**
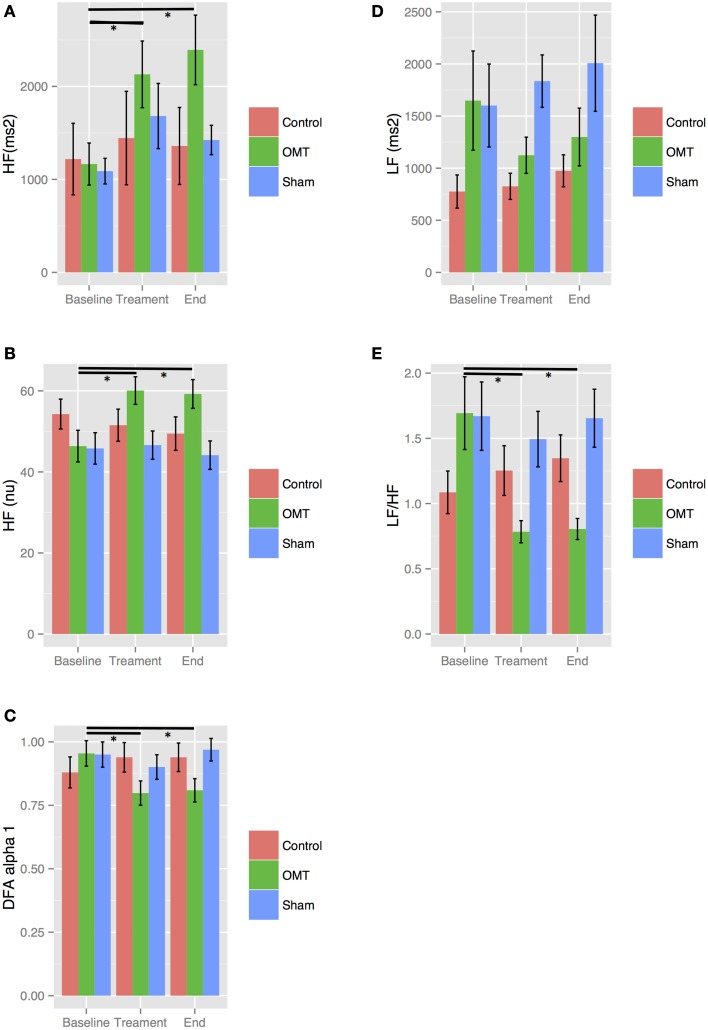
**Sensitivity analysis stratified by non-smoking**. Numbers are mean ± SEM. HF(au) **(A)**, high frequency absolute units; HF(nu) **(B)**, high frequency normalized unit; DFAα1 **(C)**, detrended fluctuation scaling exponent; LF **(D)**, low frequency; LF/HF **(E)**, low frequency/high frequency ratio. Each figure shows HRV data recorded during the three steps of the each session: baseline (5 min), treatment (15 min), end (5 min). ^*^p < 0.001 from regression mixed effect model.

Stratifying by smoking, results showed statistically significance baseline differences in the OMT group for _nu_HF, LF/HF ratio, _au_HF and DFAα1 both in smokers and non-smokers as showed in Figures 3, 4. In more details, OMT significantly changed baseline values in all HRV parameters measured (*p* < 0.01). Sham therapy and time control groups did not demonstrate any valuable change.

**Figure 5:**
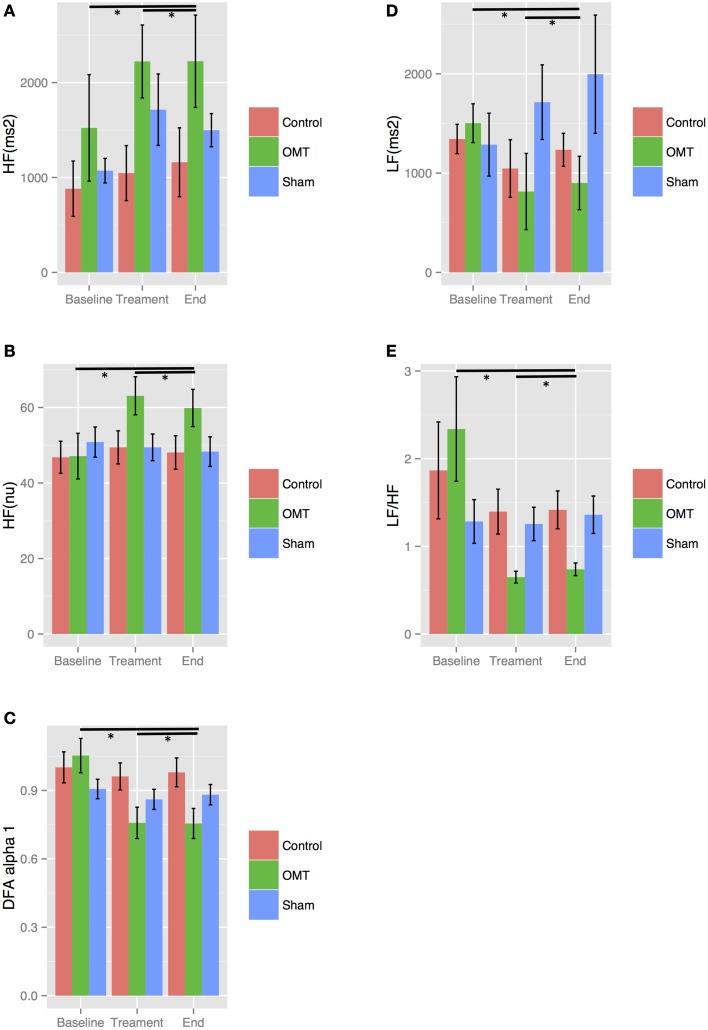
**Sensitivity analysis stratified by session 1**. Numbers are mean ± SEM. HF(au) **(A)**, high frequency absolute units; HF(nu) **(B)**, high frequency normalized unit; DFAα1 **(C)**, detrended fluctuation scaling exponent; LF **(D)**, low frequency; LF/HF **(E)**, low frequency/high frequency ratio. Each figure shows HRV data recorded during the three steps of the each session: baseline (5 min), treatment (15 min), end (5 min). ^*^p < 0.001 from regression mixed effect model.

**Figure 6:**
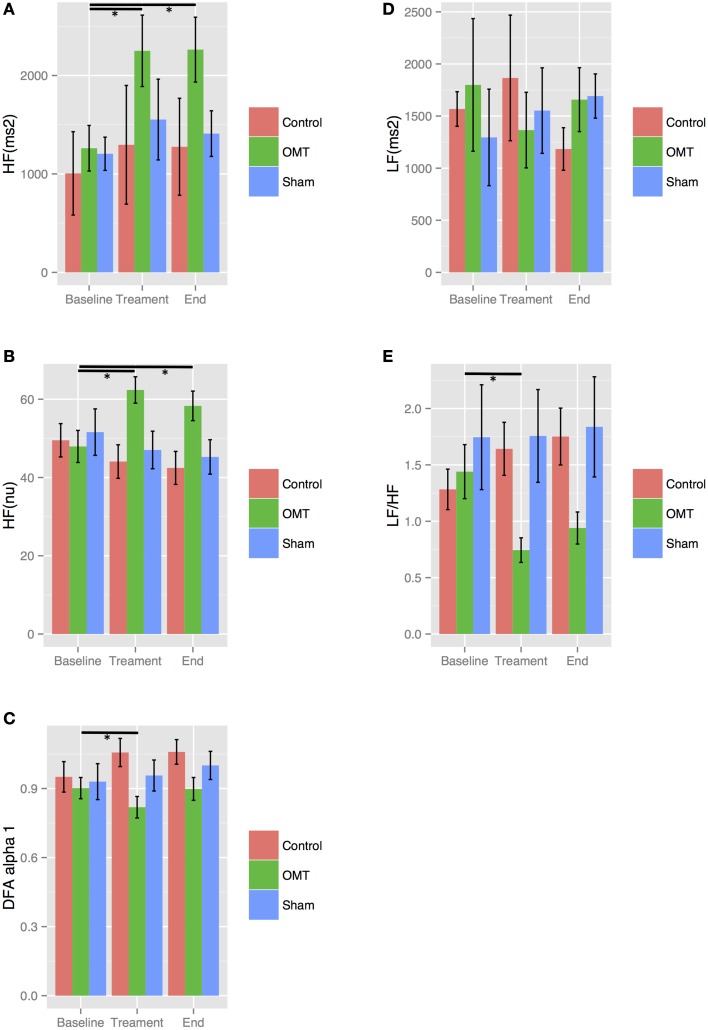
**Sensitivity analysis stratified by session 2**. Numbers are mean ± SEM. HF(au) **(A)**, high frequency absolute units; HF(nu) **(B)**, high frequency normalized unit; DFAα1 **(C)**, detrended fluctuation scaling exponent; LF **(D)**, low frequency; LF/HF **(E)**, low frequency/high frequency ratio. Each figure shows HRV data recorded during the three steps of the each session: baseline (5 min), treatment (15 min), end (5 min). ^*^p < 0.001 from regression mixed effect model. Significant differences between baseline and end periods are shown only for HF and LF/HF-values.

The first and second sessions of OMT led to an increase of _nu_HF and _au_HF as well as a decrease of _au_LF, LF/HF ratio, and DFAα1 compared to baseline values (Figures 5, 6, statistically significant at *p* < 0.01). Sham therapy and time control group did not reveal variations compared to baseline values.

### Adverse events

No adverse events were reported during the trial.

## Discussion

The present trial showed that OMT modifies ANS activity through modulating the parasympathetic functioning of healthy subjects compared to sham therapy and control groups. Statistically significant changes were revealed during the treatment period. This clinical study used a larger sample size to confirm previous findings showing that OMT can globally influence the tonic activity of ANS (Henley et al., 2008; Giles et al., 2013). However, several differences can be highlighted between former studies and this trial. Henley used an additional tilt test to evaluate HRV variations in response to an environmental stress. Then, both Henley and Giles limited the OMT intervention to anatomical areas (cervical spine and sub-occipital area) directly connected with parasympathetic and sympathetic heart rate control. The present trial used a patients' need-based treatment to improve the clinical generalizability of findings.

Interestingly, there were trend differences between smokers and non-smokers for HRV-values in the OMT group. In non-smokers, HRV-values of the final 5 min record remained unchanged compared to the HRV-values of the treatment phase. In contrast, smokers presented a difference, although not statistical significant, of the HRV-values between the final 5 min and the treatment phase, showing an influence of smoking on HRV changes after OMT. Overall, we suggest that the treatment of an osteopathic dysfunction, independently from its location, could modify autonomic activity of both smokers and non-smokers.

Interestingly, other OMT mediated improvements in clinical outcomes such as reduction of pain grade and frequency and improvement of the range of motion, may correlate to the decrease of sympathetic activity resulting from this study. As a matter of fact, parasympathetic branch of ANS has anti-inflammatory and anti-nociceptive actions. Tracey demonstrated that the release of acetylcholine by vagal endings binds to alpha-7 nicotinic receptors of macrophages selectively inhibiting pro-inflammatory cytokines production (Tracey, 2002). Sympathetic branch has instead an opposite pro-inflammatory action, potentially increasing pain grade. A 2013 study showed how interleukin-6 release, induced by norepinephrine, is mediated by β2-adrenergic receptors (Stohl et al., 2013). Clinical effects following osteopathic treatment could also be referred to the trophotropic tuning of the patient's organism, due to the shift of ANS tonic activity toward the parasympathetic functioning. According to Hess (1955), the trophotropic tuning is characterized by a decrease in frequency and an increase in amplitude of brain waves, a decrease in heart and respiratory rate, an increase in skin temperature, a decrease in muscle tension and anxiety.

Several strengths and limitations have to be considered for the present trial. No changes to methods and outcomes were applied after trial commencement. It was based on rigorous methodology, controlling for allocation, detection, sequence generation biases. Attention was paid to confounding factors like temperature, humidity of the room, smoking, and circadian rhythms. It included a large sample size and a patients' need-based treatment. Moreover, DFAα1 was taken into account as nonlinear parameter to further explore the ANS activity. Limitations are in terms of incomplete emptiness of the bladder that was not systematically asked to participants, difficulty to control for subjects emotional conditions and daily habits before the trial started.

## Conclusion

Results demonstrated that OMT produced changes of tonic activity of ANS as shown by HF, DFAα1, _au_LF, and LF/HF ratio variations. The present study focused on healthy subjects. Future studies are warranted to further explore the extent to which OMT can change ANS activity in pathological conditions and comparing its effect with usual care, hypothesizing that OMT could be used as a supportive care in addition to traditional methods.

## Author contributions

NR, GD, NM, AP, conceived the idea, drafted the first version of the paper. LC supervised the experiment, exported the data and reviewed the paper, FC run the statistical analysis, supervised the research and reviewed the paper for intellectual content. All authors approved the final version.

### Conflict of interest statement

The authors declare that the research was conducted in the absence of any commercial or financial relationships that could be construed as a potential conflict of interest.
